# Ethanol leaf extracts of *Anogeissus leiocarpus* in antioxidants and hepatotoxic effects of *Escherichia coli* O157:H7 infected Swiss Mice

**DOI:** 10.37796/2211-8039.1432

**Published:** 2024-03-01

**Authors:** Fred C. Akharaiyi, Chioma B. Ehis-Eriakha, Peter T. Olagbemide, Stephen E. Akemu

**Affiliations:** a Department of Microbiology, Edo State University Uzairue, KM 7 Auchi-Abuja Road, Edo State, Nigeria; b Department of Biological Science, Afe Babalola University, P. M. B. 5454, Ado Ekiti, Ekiti State, Nigeria

**Keywords:** Antioxidants, Acute toxicity, Bacteria, Extracts, Plant

## Abstract

**Introduction:**

Diseases caused by bacteria can be managed with medicinal plants with rightful dosage that will not affect body physiology and organs.

**Aim:**

This research aimed to evaluate the antioxidants and the effects of *Anogeisus leiocarpus* on liver function.

**Materials and methods:**

Ethanol leaf extracts were processed for antioxidants and hepatotoxic effects using animal models. Group one (negative control) was given access to water and regular feed, group two (positive control) was dosed with 107 CFU/ml of *Escherichia coli* O157:H7, and groups 3–6 were dosed with 107 *E*. *coli* O157:H7 for 3 days and treated with extract concentrations of 12, 25, 50 and 100 mg/kg bw respectively for seven days.

**Results:**

Higher ascorbic acid values in Ferric reducing antioxidant property (FRAP) and Hydroxyl radical scavenging (HRS) were recorded in the positive control (0.05 ± 0.01, 41 ± 0.05) than in the extract-treated (0.02 ± 0.14, 30 ± 0.02). Increase in DPPH (47 ± 0.1268 ± 0.05 %), Free radical scavenging property (FRAP) (0.03 ± 0.02–0.08 ± 0.14 %), and HRS (38 ± 0.14–68 ± 0.12 %) was observed in the extract. The lipid peroxidation (LPO) of the quote was 78.51 ± 2.16, GSH was 36.18 ± 3.18, and catalase was 78.42 ± 4.713. In the extract-treated, decreased values were recorded for LPO ((108.36 ± 1.12–70.19 ± 1.68 μM/g), while increased values were observed in Glutathione (GSH) (25.11 ± 2.64–33.62 ± 1.35 μM/g), and catalase (54.18 ± 2.14–60.25 ± 1.4 μM/g). The values of negative control for Aspartate aminotransferase (AST), Alanine aminotransferase (ALT), and Alkaline phosphate (ALP) were lesser than what was received in the extract treated.

**Conclusion:**

The plant’s traditional medicine usage is effective at low dosage and could be a suitable candidate for drug development which will not affect the body’s physiology and organs. The subjecting of *A*. *leiocarpus* ethanol leaf extract to antioxidants assays and its effect on liver function have further proved its value in folklore medicine.

## 1. Introduction

Microbiological diseases are a threat to humans thus the many drugs for their treatment. The urban and rural duelers use herbal remedies within their localities to solve disease problems [1]. The discovery of herbal remedies for disease management has been for ages through try and error means before civilization. It cannot be ruled that some persons died through the process of wrongful dosage or managing diseases with herbal remedies having high chemical contents. Effects of these are the lack of knowledge about the chemical contents in some plants, which affect the body’s physiology and organs. The discovery of science suggests the use of animal models for drug trials which then stopped humans from using themselves as subjects for drug test trials. Apart from the foods we eat and some environmental factors that affect the liver, synthesized chemicals and plant remedies with high amounts of chemicals from an overdose, may also result in liver dysfunction.

Hence liver dysfunction can be interpreted or reflected from biochemical physiology, in vitro and in vivo antioxidants, and histopathology, this research is therefore aimed at the antioxidants and the effects *Anogeissus leiocarpus* as a medicinal plant could have on liver function.

The plant of *A*. *leiocarpus* (DC) Guill and Perr belong to the family of Combretaceae It is commonly called the Axlewood tree. In Nigeria, this plant has some medical applications it serves in the health care system. It has been found useful to effectively treat ascaris infection, gonorrhoea, cough, clotting of blood, tuberculosis, body pain, and asthma [2]. It has also been used to treat bacterial infections [3]. In the Eastern part of Nigeria, a decoction made from the leaves is applied topically to treat the itch of psoriasis and skin infections. The powder made from the dried bark of the plant is very efficient for boils, wounds, diabetic ulcers, cysts and sores healing. The aim of this research was to determine the potency of the plant extracts for possible treatment of infections caused by some pathogenic bacteria and their liver toxicity effect.

## 2. Materials and methods

### 2.1. Plant collection for extract preparation

The leaves of *A*. *leiocarpus* were harvested from the forest around Edo State University Uzairue and were authenticated by Dr Odologie Imarhiagbe of the Department of Biological Science, Edo State University Uzairue, Nigeria. The specimen voucher was deposited in the University herbarium with the number FD 1257. The leaves were washed with tap water and rinsed severally in distilled water. It was then air-dried for three weeks at a temperature of between 25 °C and 27 °C. The leaves samples were ground to smooth powder with the aid of a mechanical grinder. One hundred grams of the powder was extracted with 250 ml of ethanol for 24 h at room temperature (27 ± 2 °C) and after which, it was filtered with Whatman Number 1 filter paper. The extract filtrate was concentrated in-vacuo and kept in a specimen bottle which was refrigerated prior to use.

### 2.2. Bacteria species

Clinical *Escherichia coli* was isolated from the diarrheic patient. The watery stool was serially diluted to 10-6 and 0.5 ml was cultured on Mac-Conkey agar and incubated at 37 °C for 24 h. The resultant bacterial colonies after incubation were purified and sub-cultured on Eosin methylene blue agar for essential cultural features. Tentative confirmation of *E*. *coli* was by colonies with a green metallic sheen. The pure isolates were Gram-stained and identified physiologically and biochemically for verification before being kept on a sterile agar slant.

### 2.3. Acute toxicity test

The methods described by the World Health Organization (WHO) in the guidelines for evaluating the efficacy and safety of herbal medicine [4] and the guidelines of the Organization of Economic Co-operation and Development (OECD) for testing chemicals [5] were adopted. Thirty-five mice consisting of both males and females were quarantined for a week to know their health status. Before the extract in vivo test for toxicity, the mice were fasted for 6 h and were divided into seven groups of five each. Group one mice were each dosed orally with 10 ml/kg body weight of normal saline, while the mice in groups’ two to seven were each dosed with 12, 25, 50, 100, 150, and 200 mg/kg body weight of extract, respectively. Toxic symptoms, according to the methods described by Lorke [6], were observed in the mice for 28 days. The Lethal Dose50 (LD50) of the extract was estimated by the method described by Miller and Tainter, [7]. The LD cut-off of the extract was at 150 mg/kg body weight. Therefore, the therapeutic extract dose for this study was between the concentrations of 12–100 mg/kg body weight.

The experimental procedures performed on the used animals were approved by the Nigerian National Health Research Ethics Committee with the assigned number of NHREC/08/2016.

### 2.4. Bacteria inoculums preparation

The bacteria isolate was purified by sub-culturing in peptone water and incubated for 18 h at 37 °C. The culture was adjusted with sterile physiological saline to match up with the McFarland standard of 10-7 colony-forming units per ml (CFU/ml).

### 2.5. Experimental animals and design

Forty-two Swiss albino mice of about 5 months and having a body weight of between 25 and 27 g were used for this study. They were accommodated for three weeks with normal rat pellets and clean tap water. Thereafter, the rats were starved of feed for 18 h but were allowed to drink water and then managed in conformity with the NIH Guide for Care and Use of Laboratory Animals. The Nigeria National Health Research Ethics Committee approval number on the performed experimental procedures is NHREC 08/2016.

Seven groups of six albino mice each were utilized for the experimental design. Group one was the negative control which got only course to rat feed and clean tap water. The mice in groups’ two to five were each infected orally in a single prescription per day with 1 ml of 10^3^ CFU/ml of *E*. *coli* for three days and treated for seven days with 12, 25, 50, and 100 mg/kg body weight of extract concentrations respectively. Thereafter, the mice in each group were sacrificed by avoiding animal cruelty, and dissected, and the liver tissues were obtained for liver function analysis and histopathology.

### 2.6. In vitro antioxidant activity assay

#### 2.6.1. Extract’s ferric-reducing antioxidant property (FRAP)

The Criteria described by Buricover and Reblova [8], were used to measure the reducing potential of the plant extract but with a few modifications. 0.1 g of extract was dissolved in 20 ml of water and filtered. 2.5 ml of the filtrate was obtained and 2.5 ml of phosphate buffer with a pH of 6.6 and 2.5 ml of potassium ferrocyanide were mixed with it. The mixture was incubated at a temperature of 50 °C. After the addition of 10 % Trichloroacetic acid, 5 ml of distilled water and 1 ml of 0.1 ferric chloride were mixed with it. The standard absorbance and that of the samples were read in a spectrophotometer at 700 nm wavelength against a reagent blank. The determinations of the reducing potential of the extract were carried out in duplicates.

#### 2.6.2. Extract’s free radical scavenging activity (DPPH)

The criteria for determining free radical scavenging activity as described by Ibanez et al. [9]; Dorman et al. [10] was used but with little modifications. From a concentration of 0.05 mg/ml in ethanol of 0.1 mm 1, 1-diphenyl 1–2 picrylhdrazyl (DPPH) radical (Sigma Aldrich, St. Louis, USA), 0.5 ml was obtained and dispensed in test tubes. .1 ml of each of the extract concentrations were added separately to each test tube and mixed up at a temperature of 27 ± 2 °C. The mixture was allowed to stand for 20 min before being placed in a spectrophotometer at a wavelength of 520 nm to read the absorbance. The absorbance value of the plant extract concentrations was expressed as mg of l-ascorbic acid per 1 g of dry plant material. In such cases, calibration was used where the extract concentrations were replaced with freshly prepared solutions of 1.6–100 mg/ml of ascorbic acid. The determinations of DPPH of the extract concentrations were replicated in triplicates. The formula below was used to calculate the percentage value of the free radical capacity of the extract concentrations:


$$\text{Percentage of DPPH scavenging activity}=\frac{\text{Ao}-\text{A}1\times 100}{\text{Ao}}$$

Ao is the control absorbance and A1 is the presence of the extract concentrations or positive control absorbance.

#### 2.6.3. Hydroxyl radical scavenging activity of the extracts

The method described by Halliwell et al. [11] was adopted but was modified. The generated hydroxyl radicals by iron-ascorbate-EDTA-H_2_O_2_ were reacted with deoxyribose to form thiobarbituric acid reactive substances (TBARS). The combined substances at low pH will give a pink chromogen if heated with trichloroacetic acid (TBA). The reaction mixture was a combination of 0.2 mM EDTA, 0.3 mM ferric chloride, 4 mM deoxyribose, 0.2 mM ascorbic acid, 2 mM H_2_O_2_ and various concentrations of extracts. The glass vials were closed tightly and incubated at 37 °C for 30 m. After which, 0.4 ml of 1 % TBA and 0.4 ml of 5 % were added to the reaction mixture and placed in a boiling water bath for 20 m. The resultant pink chromogen colour was measured against the blank samples with a spectrophotometer at 532 nm. The hydroxyl radical scavenging potential of the extract concentrations was reported as % inhibition of deoxyribose degradation. Meanwhile, ascorbic acid was used as a positive control. The determination of the hydroxyl radical scavenging activity of the extracts was replicated in triplicates. The below equation was used to calculate the inhibition percentage value:


$$\text{Inhibition }(\%)=\frac{\text{Ao}-\text{A}1\times 100}{\text{Ao}}$$

Ao was the control absorbance and A1 was the presence of the extract concentrations or positive control absorbance.

### 2.7. Assay for in vivo antioxidant activity of the extracts

The liver tissues were rinsed in 10 % physiology saline and were blended and mixed with a 1.15 % solution of potassium chloride (KCL) and 0.1 M of potassium phosphate (K_3_PO_4_) buffer with a pH of 7.4. The blended sample was thereafter centrifuged at 10000 g for an hour to obtain the supernatant required for this study. The method of Van Der Sluis et al. [12] was used to determine the amount of antioxidants by checking for lipid peroxidation (LPO) level, and the criteria of Ellman [13], was used to determine the amount of non-enzymatic antioxidant (GSH), and the enzymatic lipid peroxidation (Catalase) was determined by the method of Cohen et al. [14].

### 2.8. Estimations of biochemical

The alanine aminotransferase (ALT) and aspartate aminotransferase (ASP) activities of the extracts were performed using the methods of Bergmeyer et al. [15,16]. The assay for the alkaline phosphatase (ALP) potential of the extracts was based on colour development if serum alkaline phosphate reacts with phenolphthalein monophosphate to give phosphoric acid and phenolphthalein at alkaline pH to turn to pink colour. Colour development can be achieved with a photometric method. Bilirubin was assessed by the criteria of Watson and Rogers [17], uric acid was assessed using the criteria of Carroll et al. [18], total protein was estimated using biuret as described by Donninger et al. [19], total albumin was assessed using the criteria of Doumas et al. [20], urea was estimated using the criteria described by Fenech and Tommasini [21], estimation of total cholesterol was by the criteria description of Abel et al. [22], and creatinine was determined using the criteria of Lustgarten and Wenk [23].

### 2.9. Histopathology of liver

Pieces of liver of about 3 cm were cut from each treatment. They were rinsed in physiological saline and processed for dehydration percentage of alcohol (5 % - absolute). Clearance from traces of ethanol and water from the liver tissues was done with xylene and thereafter impregnated in liquid paraffin wax for one hour at a temperature of 60 °C. The liver tissues were immersed in liquid paraffin wax and allowed to solidify for easy sectioning. The liver tissues were sanctioned with a microtome (Bright England) at 4–5 μm. The sectioned liver tissues in a film were cut and floated in a water bath regulated at 35 °C and picked with a slide previously robbed with egg albumin. The tissues were then dewaxed with xylene, hydrated in alcohol grades (100–2 %) and cleared in xylene. Thereafter, they were stained with haematoxylin and eosin; and mounted with DPX. The histologically prepared slides were allowed to air dry, photographed and then observed with a binocular microscope for the level of damage or safety [24].

### 2.10. Statistical analysis

The obtained results are expressed as Mean ± SD. Differences were compared by One Way Analysis of Variance (ANOVA). The least significant difference (LSD) was performed for the pairwise mean comparisons, to determine the significant treatment dose at 95 % confidence level.

Consideration of values as statistically significant was at P > 0.05.

## 3. Results

The negative control tests for DPPH, FRAP, and HRS were void of in vitro antioxidant activity. However, the positive control and the extract assays contained varied values of in vitro antioxidants. In the DPPH, values of ascorbic acid of the positive control were 30 % and 40 % for the plant extract. The scavenging activity of the extract ranged from 47 to 68 %, while that of the ascorbic acid was from 36 to 56 %. This indicates that the extracts have more scavenging activity than ascorbic acid. The reducing power of the extract ranged from 0.03 to 0.08 %, while the ascorbic acid reducing capacity was from 0.06 to 0.11 %. The value comparison is significant, but the plant extracts have more reducing power. At 100 mg/ml of extract, the HRS value for plant extract was 68 % while the ascorbic acid resulted at 73 %. The hydroxyl scavenging potential of the leaf extract was also found to be better-reduced performance than the ascorbic acid (Table 1).

The in vivo antioxidant potency of the extract shows the negative control groups having lesser values in lipid peroxidation (LPO) and higher in glutathione (GSH) and catalase (CAT) than the extract-treated mice. In the extract-treated groups of mice, decreased values were recorded while increased values were recorded in GSH and CAT but on an extract concentration basis (Table 2).

The aspartate aminotransferase (AST) value in the negative control group of mice was 36.26 ± 1.43 IU/L, while in the extract concentrations of 12, 25, 50, and 100 mg/ml, it was 58.50 ± 1.52, 53.77 ± 2.68, 48.73 ± 1.34 and 46.36 ± 1.20 IU/L respectively for 12, 25, 50, and 100 mg/ml. A value of 45.2 ± 4.05 was recorded in Alanine aminotransferase (ALT) for the negative control group of mice while in the extracts, it was 57.1 ± 1.13, 54.34 ± 2.46, 52.21 ± 2.18, and 50.33 ± 1.58 respectively, alkaline phosphate (ALP) in that order was 88.20±2.53, 128.56±1.31, 125.09±1.62, 123.59 ± 2.66 and 120.26 ± 3.35 IU/L respectively (Table 3). Bilirubin content in the negative group of mice was 1.26 and in the extract concentrations of 12, 25. 50 and 100 mg/ml, it decreased from 1.78 to 1.30 mg/dL. The total albumin content in the negative group was 4.78 mg/dL and in the extracts, it decreased from 2.22 to 3.25 mg/dL. Increased protein values were also documented in the extract concentrations while a slight decrease in values was obtained in the urea and uric acid contents. Decreases in values were observed in creatinine and cholesterol contents (Table 3).

**Fig. 1:**
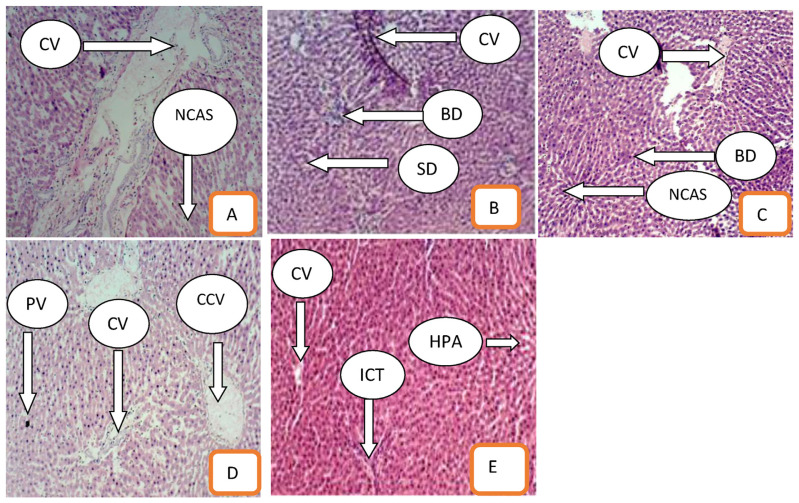
Photomicrograph of the histological liver. A (negative control), B (E. coli infected mice but treated with 12 mg/kg body weight of extract, C (E. coli infected mice but treated with 25 mg/kg body weight of extract), D (E. coli infected mice but treated with 50 mg/kg body weight of extract), E (E. coli infected mice but treated with 100 mg/kg body weight of extract). *Legend:* Central vein (CV), Normal cellular architecture (NCAS), Bile duct (BD), Sinusoids, Clear central vein (CCV), Portal vein (PV), Hepatic artery (HPA), Interlobular connective tissue (ICT).

Toxicity from the extract was not discovered in all the processed sectioned liver tissues of both the negative and positive controls. The appearance of the liver cells as observed under the microscope were in normal shape. The liver tissues either showed central vein (CV), normal cellular architecture (NCAS), bile duct (BD), sinusoids, clear central vein (CCV), portal vein (PV), hepatic artery (HPA), or interlobular connective tissue (ICT). Seen on the liver tissues that are also very important and to ascertain no toxic effect of the extract are the hepatic cells which were distinctly in cord-like fashion arrangement and separated with sinusoids (Fig. 1).

## 4. Discussion

The acute toxicity test of the plant extracts stands the LD_50_ at 150 mg/kg body weight and the therapeutic dose of extracts between 12 and 100 mg/kg body weight was therefore chosen for this study. *A*. *leiocarpus* is a plant known for its medicinal value in folklore and its analysis for the safety of human physiology and organs is of vital importance for its continuous use and probably for the development of novel drugs. Traditionally, the plant part has been used to solve arrays of ailments in African countries [25,26]. Drugs are not given without infections, and so, *E. coli* cells were injected into mice to manifest illness. The physiological changes in the mice signal infection thus, administration of the leaf extracts to evaluate the concentration (s) that will be most effective and without distortion or alteration in the mice system. By this, we were able to compare the negative with the positive control group’s values in biochemical, antioxidants, and histopathology for evaluation of the safe use of the plant remedy.

The estimated antioxidant potentials of the extract concentrations could result from the existence of phenol contents and complex inhibition properties of the extracts with enzymes. This is relatively so because the antioxidant potency of extract from plants has been associated with the phenol amounts and the inhibitory potential of the extracts with enzymes to the interplay of plant chemicals with the complex enzyme substrate.

It is proof that synthetic molecules have been used to manage free radical scavenging activity, but the side effects involved are deleterious to the body system, thus alternatives from medicinal plants are sourced because the antioxidant in plants is safe for use and has relatively low side effects [27].

We detected increased values in the LPO of the group of mice administered with the extracts and this could result from the formed free radicals and the differences that normally occurred in antioxidant garrison that perpetually caused oxidative stress. This stress is able to cause a severe decrease in the level of GSH. In previous work, Akharaiyi, and Isunu [24], have reported a similar observation.

Various factors could have been responsible for the commemorated high level of GSH after the leaf extracts treatment and such could be the progressive decrease of glutathione peroxide and the impulsive retort elicited by free-radical decrease of the cells due to exorbitant hydrogen peroxide (H2O2) production. H2O2 has been reported as a major reactive oxygen species (ROS) which leads to oxidative stress [28] and therefore required detoxification from the system for healthiness.

The increase in CAT and decrease in the LPO in the bacteria-infected mice treated with the plant extract is a reason that explains the effectiveness of the plant antioxidant. CAT can protect the liver from the physiological changes emanated by the pathogenic bacteria in the extremely responsive hydroxyl radicals. The lowered activeness of the catalase enzyme in the mice of positive control may be responsible for the hurtful aftermath in the mice due to the buildup of bacterial toxin released and hydrogen peroxide. The administered plant extract was able to enhance catalase activities and therefore stand for the prevention of the accumulated free radicals thus the liver was safeguarded from the released toxins from the pathogenic bacteria. The health condition of the studied mice’s liver cell could be the non-toxic effect of the extract. Also of importance are the contained phytochemicals and the extract concentrations used. Plants consist of a number of biologically dynamic ingredients hence they are used for treating various infectious diseases. The leaves of *A*. *leiocarpus* have been found to possess bioactive ingredients such as phenol, saponins, tannin, and flavonoids, and also not limited to other phyto-constituents [26]. This might be the reason behind the protein biosynthesis stimulation. The increase of protein in the negative control mice interprets the good level of protein contents and can hamper hepatic damage, which will lead to liver health status. The lower value of uric in the negative control below the positive control could result in adverse effects and ill health of the liver to properly perform its functions if not treated. The decreased values in the negative control as well as recorded in the creatinine, cholesterol and uric acid parameters signify no injury to the liver. However, the decrease in value in the direction of the negative control value with extract concentrations as observed in urea, total albumin, and total protein, was also the order in uric acid, cholesterol, bilirubin, and creatinine levels. These values tending to the negative control value could be resulting from the radical scavenging activity of the extract. A little level of bilirubin as a result of the breaking down of haemoglobin was noticed. This could be the rise in temperature at the bacteria disease manifestation. Albumin level was not too affected hence the liver was able to perform its normal role.

The liver was proposed in this study because it is a target of any toxicant. The liver performs the breaking down of toxins in the body and so, has to be checked against hepatotoxic agents. The liver is a sensitive organ that can be affected by chemicals and some environmental conditions to be relieved of its various functions promoting ill health and even death [29]. The representative of all liver tissues as observed under the microscope, appeared normal and either possess a central vein, bile duct, sinusoids, central vein, portal vein, hepatic artery, or interlobular connective tissue. All these are evidence of no adverse effects in using *A*. l*eicarpus* at a concentration dose of up to 100 mg/ml.

## 5. Conclusion

The present findings proved an insight that the traditional usage of this plant as a medicine is effective even at low dosages. For this, the plant could be a good candidate for drug development which will not affect the body’s physiology and organs. The subjecting of *A*. *leiocarpus* ethanol leaf extracts to antioxidants assays and its effect on liver function have further proved the medicinal plant’s value in folklore medicine.

## Figures and Tables

**Table 1:** In vitro antioxidant quality of A. leiocarpus.

Group	DPPH (%)		FRAP (%)		HRS (%)	
		
	Ascorbic acid	*A. leiocarpus*	Ascorbic acid	*A*. *leiocarpus*	Ascorbic acid	*A*. *leiocarpus*
−ve control	0 ±0.00	0 ±0.00	0 ±0.00	0 ±0.00	0 ±0.00	0 ±0.00
+ve control	30 ±0.02	40 ±0.06	0.05 ±0.01	0.02 ±0.14	41 ±0.05	30 ±0.02
12 mg/ml	36 ±0.05	47 ±0.12	0.06 ±0.05	0.03 ±0.02	47 ±0.07	38 ±0.14
25 mg/ml	42 ±0.03	50 ±0.01	0.06 ±0.02	0.04 ±0.03	56 ±0.00	39 ±0.03
50 mg/ml	51 ±0.10	60 ±0.07	0.08 ±0.05	0.04 ±0.11	63 ±0.14	61 ±0.12
100 mg/ml	56 ±0.04	68 ±0.05	0.11 ±0.13	0.08 ±0.14	73 ±0.11	68 ±0.12

**Table 2:** In vivo antioxidant quality of A. leiocarpus.

Group	LPO (μM/g)	GSH (μM/g)	Catalase (μM/g)
−VE control	78.51 ±2.16^a^	36.18 ±3.18^a^	78.42 ±4.713^a^
12 mg/ml	108.36 ±1.12^bc^	25.11 ±2.64^c^	54.18 ±2.14^c^
25 mg/ml	90.45 ±1.11^c^	27.14 ±2.40^bc^	57.24 ±1.63^b^
50 mg/ml	85.18 ±3.20^b^	30.54 ±1.53^b^	58.31 ±1.26^b^
100 mg/ml	80.19 ±1.68^b^	33.62 ±1.35^a^	60.25 ±1.4^b^

Values are mean ±standard deviations of triplicate determination.

The values with different superscript (a–c) per column are significantly different.

**Table 3:** Anogeissus leiocarpus ethanol leaf extract on liver functions of mice.

Group	AST	ALT	ALP	Bilirubin	Total albumin	Total protein	Urea	Uric acid	Creatinine	Cholesterol
									
	(IU/L)	(IU/L)	(IU/L)	(mg/dL)	(mg/dL)	(mg/dL)	(mg/dL)	(mg/dL)	(mg/dL)	(mg/dL)
Control (−)	36.26 ±1.43	45.2 ±4.05	88.20 ±2.53	1.24 ±0.13	4.78 ±0.11	6.65 ±0.45	18.15 ±1.62	5.10 ±0.41	1.25 ±0.27	112.51 ±0.310
12 mg/kgbw	58.50 ±1.52	57.1 ±1.13 128.56 ±1.31	1.78 ±0.24	2.22 ±0.32	5.16 ±0.32	21.54 ±0.42	8.22 ±0.09	1.78 ±0.44	156.03 ±6.05	
25 mg/kgbw	53.77 ±2.68	54.34 ±2.46	125.09 ±1.62	1.57 ±0.34	2.75 ±0.20	5.32 ±0.15	21.63 ±0.34	8.09 ±0.65	1.75 ±0.20	145.33 ±1.04
50 mg/kgbw	48.73 ±1.34	52.21 ±2.18	123.59 ±2.66	1.54 ±0.10	3.13 ±0.08	5.25 ±0.00	21.72 ±0.20	7.56 ±1.00	1.73 ±0.32	138.11 ±0.22
100mg/kgbw	46.36 ±1.20	50.33 ±1.58	120.26 ±3.35	1.30 ±0.18	3.25 ±0.16	5.46 ±0.20	21.76 ±1.15	7.32 ±0.07	1.51 ±0.18	133.21 ±0.13
